# Study on the effectiveness of a community hypertension management model based on home smart blood pressure monitoring using IoT technology

**DOI:** 10.3389/fpubh.2024.1428310

**Published:** 2024-08-02

**Authors:** Cheng Li, Shuhao Fan, Hui Li

**Affiliations:** ^1^Centre for Health Management and Policy Research, School of Public Health, Cheeloo College of Medicine, Shandong University, Jinan, China; ^2^NHC Key Lab of Health Economics and Policy Research (Shandong University), Jinan, China; ^3^Quality Management Department, Shandong Cancer Hospital and Institute, Shandong First Medical University and Shandong Academy of Medical Sciences, Jinan, China

**Keywords:** hypertension, difference-in-differences model, mediating effects, Internet of Things, smart blood pressure monitoring

## Abstract

**Background:**

Hypertension is rapidly increasing in China, but control rates are significantly low. There is a pressing need for effective management models in primary community health settings.

**Methods:**

In April 2023, 459 patients from six communities in Jinan City were enrolled using a multi-stage random sampling method and assigned to either a control group comprising 243 participants or an intervention group comprising 216 participants. The control group received standard hypertension care, whereas the intervention group participated in a novel IoT-based remote blood pressure monitoring program for 6 months. Data collection was conducted through detailed questionnaire surveys, cloud platform records, and community management records, both before and after the intervention period. The study employed difference-in-differences (DID) and mediation effect models to assess the effects of the IoT-based management model.

**Results:**

The DID model demonstrated that the intervention significantly reduced systolic blood pressure by 9.883 mm Hg and diastolic blood pressure by 6.553 mm Hg. The mediation effect model showed that the frequency of blood pressure measurement and attitudes and beliefs toward hypertension treatment had mediating effects, accounting for 5.82 and 8.07% of the total effect, respectively. The heterogeneity analysis revealed significant regional differences: rural residents experienced a greater decrease in systolic and diastolic blood pressures by 14.85 mm Hg and 6.591 mm Hg, respectively, compared to urban residents, whose diastolic pressure decreased by 6.046 mm Hg.

**Recommendations:**

It is advisable to develop differentiated hypertension management strategies tailored to specific regional needs to promote the deep integration and widespread application of smart blood pressure monitoring technology. Enhancing patient awareness and capabilities in managing their health condition is crucial for improving the blood pressure control level among community hypertension patients.

## 1 Introduction

With the transition in the disease spectrum from acute to chronic diseases and the intensification of population aging, chronic disease management has become a globally significant public health issue (1). Hypertension is among the most prevalent chronic diseases worldwide and is the most critical risk factor for cardiovascular and cerebrovascular diseases (2–4). Hypertensive patients account for 45% of deaths from ischemic heart disease and 51% from cerebrovascular diseases (5). Effectively controlling blood pressure can significantly reduce the risks of stroke, coronary heart disease, congestive heart failure, and all-cause mortality. A decrease of 10 mmHg in systolic blood pressure can lower the risk of stroke and coronary heart disease events by approximately 40 and 30%, respectively (6, 7). As a chronic and irreversible condition, the improvement of prognosis in hypertension depends on effective blood pressure management and the prevention and control of cardiovascular and cerebrovascular complications. The community is the primary setting for such management, yet to date, there is still no universally accepted, scientific, effective, and replicable model for community-based management of chronic diseases in the older adult. How to keep blood pressure within an appropriate range presents a common challenge in the global treatment of hypertension. Research reports indicate that in surveys conducted across 115 communities in 12 provinces of China, the awareness rate of hypertension was 41.6%, the treatment rate was 34.4%, and the control rate was only 8.2%, showing that blood pressure control rates remain poor (8).

In recent years, the integration of information technology into the management of chronic diseases and the transformation of these management models toward digitization and intelligence has emerged as a focal point of academic research (9). The development of “Internet of Things (IoT) + Healthcare” presents a cost-effective approach to supporting self-management behaviors in hypertension patients, boasting significant scalability (10). It is widely acknowledged that self-monitoring of blood pressure (SMBP) can lead to reductions in blood pressure levels, though the exact mechanisms remain to be clarified (11, 12). Furthermore, SMBP on its own does not suffice for achieving clinically meaningful blood pressure control without the concurrent implementation of appropriate interventions by healthcare professionals (13). Thus, identifying the mediating factors within the hypertension intervention process and quantifying their impact are of paramount importance. The initial groundwork of this study included providing patients with smart blood pressure monitors, leveraging cloud platforms for remote monitoring of blood pressure, and fostering patients’ attitudes and beliefs toward hypertension treatment through online doctor-patient communication and regular follow-up guidance when anomalies are detected, all while offering timely medical advice and support. Building on this foundation, this article aims to assess the application effectiveness of home smart blood pressure monitoring based on IoT technology within a community hypertension management model. It further seeks to quantitatively analyze the mechanisms by which home smart blood pressure monitoring impacts community residents’ blood pressure levels, exploring the various channels involved. It also examines whether there are heterogeneous differences under different regional constraints, subsequently proposing key strategies for optimizing community-based hypertension management in China.

## 2 Materials and methods

### 2.1 Study subjects

#### 2.1.1 Community selection

Our study utilized a multi-stage random sampling method, employing simple random sampling via a random number table at each stage to ensure rigorous randomization and prevent any selection bias. To ensure the diversity and representativeness of the initial sample, two districts, Licheng and Tianqiao, were initially selected from among Jinan City’s 12 districts using simple random sampling. Subsequently, from a total of 138 communities in these districts, six communities were chosen using the same sampling approach. The selected communities are as follows: Daqiao, Sangzi, Lingang, Dikou, Baohua, and Dongfeng. The criteria for selecting these communities included: having the intention to improve the management level of hypertension prevention and control within the jurisdiction; undertaking the management of hypertension patients as part of the basic public health services in the area; possessing a solid foundation for the management of community hypertension patients, capable of maintaining effective communication with patients to ensure the smooth progression of the project; including a population of hypertension patients: having over 100 hypertension patients after excluding those who do not meet the project’s inclusion criteria; having at least two dedicated hypertension management personnel; having a computer with access to the public internet.

#### 2.1.2 Selection criteria and number of hypertension patients within the community

Each community health service institution screened all eligible patients from among those managed for hypertension, based on inclusion and exclusion criteria. The list of patients was provided by community doctors, and the specific inclusion and exclusion criteria for the study subjects are presented in Table 1. After compiling a list of eligible patients from three urban and three rural communities, simple random sampling using a random number table method was applied to hypertensive patients under management in each community. The sample size calculation Equation (1) is as follows:


(1) $$n=\frac{{\left(Z_{\alpha }+Z_{\beta }\right)}^{2}}{\delta^{2}}*2\sigma^{2}$$

where *α* = 0.05, $Z_{\alpha }$ = 1.96; test efficacy is 0.8, $Z_{\beta }$ = 0.84; σ represents the standard deviation and δ represents the difference, in this case σ = 20 and δ = −4 from the pre-experiment. Given this calculation, we aimed to recruit approximately 196 participants per group, totaling 392. However, to accommodate potential attrition (estimated at 20%), we increased our target enrollment to approximately 240 participants per group, leading to a total of 480. Ultimately, a total of 459 subjects fully participated in the research, with 243 in the control group and 216 in the intervention group. All study participants signed informed consent forms.

**Table 1:** Inclusion and exclusion criteria for study participants.

Inclusion criteria	Exclusion criteria
(1) Aged over 35 years; (2) on the hypertension register in primary health center; (3) not already taking more than 3 anti-hypertensive agents; (4) on a stable dose of current anti-hypertensive medication for at least 4 weeks prior to trial entry.	(1) Orthostatic hypotension (20 mmHg or more systolic drop after standing for 1 min, in order to avoid adverse events); (2) diagnosed atrial fibrillation (automated monitors not validated); (3) unwilling to self-monitor; (4) female participant who is pregnant, lactating or planning pregnancy during the trial (management of essential hypertension in pregnancy is different); (5) the partner or spouse of an individual already randomized in the trial (to avoid clustering within families); (6) Chronic Kidney Disease (CKD) grade four or worse; (7) any grade of CKD with proteinuria (may have different BP targets); (8) participants who have participated in another research trial involving anti-hypertensive medication in the past 4 weeks.

### 2.2 Intervention project implementation method

This project commenced in April 2023 and spanned a duration of 6 months, during which patient management was systematically divided into an intervention group and a control group. The intervention group implemented a community hypertension management model based on home remote blood pressure monitoring with IoT technology, while the control group followed the routine community hypertension management. The basic management methods and tasks are as follows.

Control group: Management was carried out according to the conventional model for hypertension patients, with community health service institution doctors completing biannual follow-ups for each managed individual. Additionally, if patients had needs, they could visit or consult the community health service institution, receiving care according to regular management and treatment models.

Intervention group: (1) Distribution of smart blood pressure monitors and organization of training for patients on self-measurement of blood pressure (including the method of measurement, timing, and frequency requirements), establishment of personal management accounts, and linkage to remote electronic blood pressure monitors. This task was conducted simultaneously with the pre-intervention patient survey by the survey team. (2) Remote blood pressure monitoring: Patients measured their blood pressure at home, with results automatically uploaded and stored in the cloud, generating a list of blood pressure readings and trends that could be accessed by both patients and healthcare providers at any time. Automatic alerts were sent to both healthcare providers and patients when measurements exceeded pre-set abnormal thresholds, prompting doctors to promptly respond to these alerts by actively communicating with patients to identify causes and adjust diagnostic and treatment plans. (3) Regular follow-up guidance: For patients without abnormal alerts, doctors regularly (monthly) reviewed patients’ blood pressure measurements and trends, communicating with patients online or offline as needed. If indications for medication adjustments were present, patients received information for adjusting their medication regimens, and if patients experienced changes in their condition or indications for a follow-up, reminders and suggestions for further check-ups were provided. (4) Online doctor-patient communication: Patients and their families could establish an online communication platform with managing doctors through a WeChat public account, where managing doctors could provide guidance on patient inquiries. (5) Intervention records: Regardless of the form of follow-up and management conducted, basic records of the work were maintained in the app.

This project utilizes the “Huiqing’an Brand Smart Blood Pressure Monitor (Model ZNYL-102),” equipped with GPRS wireless data transmission capability. It is the only smart blood pressure monitoring system in China that has been certified through clinical trial research. It is also the sole product to receive a patent for smart blood pressure technology and is the only one designated by the Shandong Society for Preventive Medicine as a product for remote hypertension monitoring. The system functions as a “smart medical terminal device,” enabling hypertension patients to measure their blood pressure with a device that features built-in memory and real-time data transmission capabilities. It provides services and health management through the “Public Health Cloud Platform,” catering to both users and institutions. Blood pressure data is uploaded in real time to an online data management platform, which generates reports on the patient’s blood pressure trends over time. If blood pressure exceeds predefined thresholds, the platform sends alerts to the patient and their family, as well as to community healthcare providers managing the patient’s hypertension. This enables physicians to communicate based on monitoring results, enhance follow-up consultations, adjust medication plans, and offer effective interventions. The community hypertension management platform based on home remote blood pressure monitoring utilizes mobile internet and smart technology to transcend spatial and geographical limitations, facilitating data sharing among doctors, patients, families, and the platform.

### 2.3 Data collection

The data were derived from three sources: First, pre- and post-intervention self-administered questionnaire surveys of patients cover aspects such as gender, age, education level, economic status, treatment attitudes, and belief scales, along with onsite physical examination data, including blood pressure, body measurements, weight, and waist circumference. Second, cloud platform record data during the 6 months of patient intervention include each blood pressure monitoring value, time points, number of alert reports, and the number of communications on the patient-healthcare provider platform. Third, management records from the community health service center contain follow-up records, outpatient visits, and telephone communications. Among these, the Cronbach’s *α* coefficient for the hypertension treatment attitudes and beliefs scale was 0.70, indicating good reliability of the questionnaire.

Furthermore, to ensure the completeness and authenticity of the data to the greatest extent, this study implemented strict quality control at every stage, including research design, survey preparation, field investigation, data organization, and analysis.

### 2.4 Statistical methods

Statistical analysis was conducted using STATA 17.0 software. Quantitative data that conformed to a normal distribution were denoted as $\bar{x}\pm s$ , and comparisons between groups were made using independent sample t-tests. For non-normally distributed data, medians and interquartile ranges were presented, with non-parametric tests used for inference. Categorical data were expressed as counts (*n*) and percentages (%), with comparisons between groups conducted using the $\chi^{2}$ test. The Difference-in-Differences (DID) model was employed to evaluate the effect of the smart blood pressure monitoring intervention project on the blood pressure of community residents. The significance level was set at *ɑ* = 0.05. The basic setup of the DID model is as follows:


(2) $$Y_{\mathrm{it}}=\beta_{0}+\beta_{1}DID_{\mathrm{it}}+\beta_{2}Time_{t}+\beta_{3}\mathrm{Grou}p_{i}+\varphi X_{\mathrm{it}}+\varepsilon_{\mathrm{it}}$$

In the model, $Y_{\mathrm{it}}$ represents the dependent variables, which include systolic and diastolic blood pressure. The core explanatory variable in this paper is $DID_{\mathrm{it}}$ , defined as $DID_{\mathrm{it}}=Time_{t}*\mathrm{Grou}p_{i}$ . Here, $\mathrm{Grou}p_{i}$ denotes the group variable, where if individual i is in the intervention group, which utilizes a community hypertension management model based on IoT technology for home remote blood pressure monitoring, $\mathrm{Grou}p_{i}$ is set to 1. Conversely, if individual *i* is in the control group, which follows conventional community hypertension management, $\mathrm{Grou}p_{i}$ is set to 0. The variable $Time_{t}$ represents the time period, with $Time_{t}=0$ for the baseline period prior to implementation and $Time_{t}=1$ for the final period post-implementation. $X_{\mathrm{it}}$ encompasses observable control variables, and $\varepsilon_{\mathrm{it}}$ denotes the random error term. The coefficient of the interaction term, $DID_{\mathrm{it}}$ , noted as $\beta_{1}$ , is of primary interest as it quantifies the net effect of the smart blood pressure monitoring intervention on the blood pressure levels of residents. This $DID_{\mathrm{it}}$ estimate captures the isolated impact attributable to the intervention.

Furthermore, this paper introduces mediating variables to further test whether the smart blood pressure monitoring intervention project reduces community residents’ blood pressure by enhancing the frequency of blood pressure measurement or the patients’ treatment attitudes and beliefs. The model is constructed as follows:


(3) $$y_{\mathrm{it}}=a_{0}+a_{1}DID_{\mathrm{it}}+a_{3}Time_{t}+a_{4}\mathrm{Grou}p_{i}+a_{5}X_{\mathrm{it}}+\varepsilon_{\mathrm{it}}$$

(4) $$\mathrm{Me}d_{\mathrm{it}}=b_{0}+b_{1}DID_{\mathrm{it}}+b_{3}Time_{t}+b_{4}\mathrm{Grou}p_{i}+b_{5}X_{\mathrm{it}}+\varepsilon_{\mathrm{it}}$$

(5) $$y_{\mathrm{it}}=c_{0}+c_{1}DID_{\mathrm{it}}+c_{2}\mathrm{Me}d_{\mathrm{it}}+c_{3}Time_{t}+c_{4}\mathrm{Grou}p_{i}+c_{5}X_{\mathrm{it}}+\varepsilon_{\mathrm{it}}$$

In Equations (3−5), Med represents the mediating variables of the study, including the frequency of blood pressure measurement (Frequency) and treatment attitudes and beliefs (TAB). The definitions of all other variables remain consistent with those outlined in Equation (2). The product of the model coefficients $b_{1}c_{2}$ represents the mediating effect in this study. It is related to the direct effect $c_{1}$ and the total effect $a_{1}$ through the relationship described by Equation (6):


(6) $$a_{1}=c_{1}+b_{1}c_{2}$$

## 3 Results

### 3.1 Comparison of baseline data between control and intervention groups

As shown in Table 2, there were no significant statistical differences in gender, age, marital status, education level, economic status, and family history between the intervention and control groups at baseline (*p* > 0.05). This indicates that the selection of participants in both the intervention and control groups was well-balanced prior to the intervention, ensuring comparability and providing a solid foundation for the subsequent analysis.

**Table 2:** Comparison of baseline data between control and intervention groups.

Characteristics	Total (*n* = 459)	Control group (*n* = 243)	Intervention group (*n* = 216)	*χ* ^2^	*p*
Gender				0.332	0.564
Female	257 (55.99)	133 (54.73)	124 (57.41)		
Male	202 (44.01)	110 (45.27)	92 (42.59)		
Age				0.079	0.778
65 and above	347 (75.60)	185 (76.13)	162 (75.00)		
Below 65	112 (24.40)	58 (23.87)	54 (25.00)		
Marital status				0.016	0.901
Single	67 (14.60)	35 (14.40)	32 (14.81)		
Married	392 (85.40)	208 (85.60)	184 (85.19)		
Education level				1.430	0.232
Middle school and below	333 (72.55)	182 (74.90)	151 (69.91)		
High school and above	126 (27.45)	61 (25.10)	65 (30.09)		
Economic status				0.399	0.527
Poor	82 (17.86)	46 (18.93)	36 (16.67)		
Good	377 (82.14)	197 (81.07)	180 (83.33)		
Family history				0.133	0.715
Yes	253 (55.12)	132 (54.32)	121 (56.02)		
No	206 (44.88)	111 (45.68)	95 (43.98)		

### 3.2 Evaluation of the intervention project’s implementation effectiveness

As shown in Table 3, prior to the intervention, both the control and intervention groups were balanced in terms of systolic and diastolic blood pressure (*p* > 0.05), allowing for comparability. After intervention and without controlling for any confounding factors, there was no significant change in systolic blood pressure among residents in the control group, while systolic blood pressure in the intervention group decreased from 151 to 141. Compared to the control group, the median difference in systolic blood pressure for the intervention group was −8.00 mmHg (*p* < 0.01). Similarly, without controlling for any confounding factors, diastolic blood pressure increased from 86 to 87 among residents in the control group, whereas in the intervention group, it decreased from 89 to 82. Compared to the control group, the median difference in diastolic blood pressure for the intervention group was −5.00 mmHg, and this difference was statistically significant (*p* < 0.01).

**Table 3:** Changes in residents’ blood pressure pre- and post-intervention.

Time	Variable	Group	*n*	Median (*P*_25_, *P*_75_)	Md	*Z*	*p*
Pre-intervention	SBP	Control	243	149 (135, 165)	2	−1.196	0.232
		Intervention	216	151 (138, 169)			
	DBP	Control	243	86 (78, 93)	3	−1.512	0.130
		Intervention	216	89 (78, 96)			
Post-intervention	SBP	Control	243	149 (138, 160)	−8	−4.116	0.000^***^
		Intervention	216	141 (130, 155)			
	DBP	Control	243	87 (80, 95)	−5	−4.843	0.000^***^
		Intervention	216	82 (75, 89)			

SBP, Systolic Blood Pressure; DBP, Diastolic Blood Pressure; Md, Median difference; *Z*, Mann–Whitney test statistic *z* value.

To better eliminate the influence of confounding factors and to reflect the causal judgment between the intervention project and its implementation outcomes, this study utilized a difference-in-differences (DID) model for data analysis. After adjusting for factors such as age, gender, marital status, Education level, economic status, and Family history, the implementation of the smart blood pressure monitoring intervention project resulted in a significant reduction in systolic blood pressure by 9.883 mm Hg and diastolic blood pressure by 6.553 mm Hg among hypertensive patients compared to changes in the control group (*p* < 0.05), as shown in Table 4. This indicates that the community hypertension management model, based on home smart blood pressure monitoring with IoT technology, significantly improved the control rate of hypertension among community patients, with both systolic and diastolic blood pressures showing a decrease.

**Table 4:** DID evaluation of the impact of the intervention project on residents’ blood pressure.

Variable	(1)	(2)
	Systolic blood pressure	Diastolic blood pressure
DID	−9.883^***^	−6.553^***^
	(−3.71)	(−4.15)
Time	−0.844	0.794
	(−0.47)	(0.74)
Group	2.483	1.645
	(1.25)	(1.38)
Age	−5.165^***^	5.312^***^
	(−3.27)	(5.32)
Gender	1.802	2.796^***^
	(1.30)	(3.30)
Marital status	−1.026	1.321
	(−0.54)	(1.28)
Education level	−4.798^***^	−2.433^***^
	(−3.11)	(−2.73)
Economic status	3.716^**^	1.532
	(2.18)	(1.50)
Family history	−0.468	−0.934
	(−0.34)	(−1.16)
_cons	150.7^***^	82.83^***^
	(57.01)	(55.51)
*N*	918	918
*R* ^2^	0.067	0.086

*t*-values after considering clustering standard errors are in parentheses; ***, **, * indicate significant correlations at the 1, 5, and 10% levels, respectively; same for the latter table.

### 3.3 Mediating effect analysis

#### 3.3.1 Mediating effect analysis of blood pressure measurement frequency

On one hand, the smart blood pressure monitoring intervention project integrates the advantages of smart technology and health management, reducing the cost and barriers to blood pressure management, addressing the deficiencies of traditional blood pressure management methods, enhancing the efficiency of residents’ health management, and providing community residents with convenient blood pressure monitoring services. On the other hand, through remote blood pressure monitoring, regular follow-up guidance, and online doctor-patient communication, smart blood pressure monitoring leverages big data and cloud computing technology for rapid integration and analysis of health data. This reduces the information asymmetry problem existing between doctors and patients, enhances the effectiveness of blood pressure management, and promotes the improvement of community residents’ health levels. Based on this, the present study empirically explores the extent to which the smart blood pressure monitoring intervention project reduces systolic and diastolic blood pressure levels by strengthening the frequency of blood pressure measurement among community residents.

This study follows a stepwise estimation procedure for testing mediating effects. Initially, it examines the overall impact of the smart blood pressure monitoring intervention on community residents’ blood pressure levels. Results shown in columns (1) and (4) of Table 5 indicate that the intervention significantly reduced both systolic and diastolic blood pressure levels at the 1% significance level. Subsequently, the mediating variable, frequency of blood pressure measurement, was incorporated into Model (4) for regression analysis. The results in columns (2) and (5) of Table 5 demonstrate that the impact coefficient of the intervention on the frequency of blood pressure measurement is positive at the 5% significance level. This confirms the robustness of smart blood pressure monitoring in increasing the frequency of blood pressure measurement. Lastly, the study verifies whether the frequency of blood pressure measurement acts as a mediating factor in reducing blood pressure levels among community residents through the intervention. As indicated in columns (3) and (6) of Table 5, after incorporating the mediating variable into the econometric model (5), the estimated coefficient $c_{2}$ is significant at the 5% statistical level, suggesting that the frequency of blood pressure measurement significantly mediates the relationship. At the same time, the DID coefficient $c_{1}$ of the smart blood pressure monitoring intervention is significant at the 1% statistical level, and with $c_{1}$ being less than $a_{1}$ , it suggests that the frequency of blood pressure measurement partially mediates the relationship, serving as an indirect channel through which smart blood pressure monitoring reduces the blood pressure levels of community residents. Further calculations reveal that the mediating effect of blood pressure measurement frequency ( $b_{1}c_{2}$ ) accounts for 5.82% of the total effect ( $a_{1}$ ).

**Table 5:** Mediating effect analysis of blood pressure measurement frequency.

Variable	(1)	(2)	(3)	(4)	(5)	(6)
	Systolic blood pressure	Frequency	Systolic blood pressure	Diastolic blood pressure	Frequency	Diastolic blood pressure
DID	−9.883^***^	0.459^**^	−9.308^***^	−6.553^***^	0.459^**^	−6.185^***^
	(−3.71)	(2.57)	(−3.46)	(−4.15)	(2.57)	(−3.87)
Frequency			−1.253^**^			−0.804^***^
			(−2.47)			(−2.68)
Time	−0.844	0.189	−0.606	0.794	0.189	0.946
	(−0.47)	(1.46)	(−0.34)	(0.74)	(1.46)	(0.90)
Group	2.483	0.127	2.642	1.645	0.127	1.747
	(1.25)	(0.90)	(1.34)	(1.38)	(0.90)	(1.48)
Age	−5.165^***^	−0.0816	−5.267^***^	5.312^***^	−0.0816	5.247^***^
	(−3.27)	(−0.77)	(−3.35)	(5.32)	(−0.77)	(5.26)
Gender	1.802	0.117	1.949	2.796^***^	0.117	2.890^***^
	(1.30)	(1.28)	(1.41)	(3.30)	(1.28)	(3.42)
Marital status	−1.026	0.213	−0.759	1.321	0.213	1.493
	(−0.54)	(1.55)	(−0.40)	(1.28)	(1.55)	(1.47)
Education level	−4.798^***^	0.318^***^	−4.399^***^	−2.433^***^	0.318^***^	−2.177^**^
	(−3.11)	(2.97)	(−2.85)	(−2.73)	(2.97)	(−2.46)
Economic status	3.716^**^	−0.252^**^	3.399^**^	1.532	−0.252^**^	1.329
	(2.18)	(−2.26)	(1.99)	(1.50)	(−2.26)	(1.30)
Family history	−0.468	−0.106	−0.601	−0.934	−0.106	−1.019
	(−0.34)	(−1.13)	(−0.44)	(−1.16)	(−1.13)	(−1.27)
_cons	150.7^***^	5.101^***^	157.1^***^	82.83^***^	5.101^***^	86.93^***^
	(57.01)	(27.48)	(41.35)	(55.51)	(27.48)	(38.62)
*N*	918	918	918	918	918	918
*R* ^2^	0.067	0.074	0.074	0.086	0.074	0.094

#### 3.3.2 Mediating effect analysis of treatment attitudes and beliefs

The smart blood pressure monitoring intervention project, merging advanced smart technology with comprehensive health education, has significantly streamlined the blood pressure monitoring process and substantially enhanced community residents’ awareness of the importance of hypertension management. This heightened awareness further motivates residents to actively participate in blood pressure management, optimizing treatment outcomes. Moreover, the application of smart blood pressure monitoring fosters ongoing communication and interaction between doctors and patients, enabling doctors to promptly track changes in patients’ blood pressure and adjust treatment plans accordingly. Such interactions not only bolster health awareness among community residents but also provide them with more personalized and precise treatment recommendations, thereby effectively facilitating blood pressure control (13, 14). Based on this, the present study delves into how the smart blood pressure monitoring intervention project lowers blood pressure levels by strengthening community residents’ attitudes and beliefs regarding hypertension treatment.

The analysis of the mediating effect of treatment attitudes and beliefs (TAB) continues to adhere to the stepwise estimation testing procedure. Initially, the study examines the overall impact of the smart blood pressure monitoring intervention on community residents’ blood pressure levels. The results shown in columns (1) and (4) of Table 6 indicate that the smart blood pressure monitoring intervention significantly lowered both systolic and diastolic blood pressure levels of community residents at the 1% level. Subsequently, the mediating variable, treatment attitudes and beliefs, was incorporated into Model (4) for regression analysis. The findings in columns (2) and (5) of Table 6 demonstrate that the effect coefficient of the smart blood pressure monitoring intervention on the treatment attitudes and beliefs of community residents is positive at the 1% significance level. This confirms that, as theorized, the impact of smart blood pressure monitoring in enhancing residents’ treatment attitudes and beliefs is robust. Lastly, the study verifies whether treatment attitudes and beliefs act as a mediating factor in reducing blood pressure levels among community residents through the intervention. Columns (3) and (6) of Table 6 reveal that after including the mediating variable of treatment attitudes and beliefs into the econometric model (5), the estimated coefficient $c_{2}$ is significant at the 5% statistical level, indicating that treatment attitudes and beliefs significantly mediate the relationship. Simultaneously, the DID coefficient $c_{1}$ of the smart blood pressure monitoring intervention is significant at the 1% level, and with $c_{1}$ being less than $a_{1}$ , it suggests that treatment attitudes and beliefs partially mediate the relationship, serving as an indirect pathway through which smart blood pressure monitoring reduces the systolic and diastolic blood pressure levels of community residents. Further calculations reveal that the mediating effect of treatment attitudes and beliefs ( $b_{1}c_{2}$ ) accounts for 8.07% of the total effect ( $a_{1}$ ).

**Table 6:** Mediating effect analysis of treatment attitudes and beliefs.

Variable	(1)	(2)	(3)	(4)	(5)	(6)
	Systolic blood pressure	TAB	Systolic blood pressure	Diastolic blood pressure	TAB	Diastolic blood pressure
DID	−9.883^***^	1.792^***^	−9.354^***^	−6.553^***^	1.792^***^	−6.064^***^
	(−3.71)	(3.22)	(−3.48)	(−4.15)	(3.22)	(−3.85)
TAB			−0.296^**^			−0.273^***^
			(−2.11)			(−2.83)
Time	−0.844	0.0370	−0.833	0.794	0.0370	0.804
	(−0.47)	(0.09)	(−0.46)	(0.74)	(0.09)	(0.76)
Group	2.483	−0.649	2.292	1.645	−0.649	1.468
	(1.25)	(−1.49)	(1.15)	(1.38)	(−1.49)	(1.23)
Age	−5.165^***^	−0.0201	−5.171^***^	5.312^***^	−0.0201	5.307^***^
	(−3.27)	(−0.06)	(−3.28)	(5.32)	(−0.06)	(5.30)
Gender	1.802	0.0714	1.823	2.796^***^	0.0714	2.816^***^
	(1.30)	(0.25)	(1.31)	(3.30)	(0.25)	(3.33)
Marital status	−1.026	−0.124	−1.063	1.321	−0.124	1.288
	(−0.54)	(−0.31)	(−0.56)	(1.28)	(−0.31)	(1.24)
Education level	−4.798^***^	0.335	−4.699^***^	−2.433^***^	0.335	−2.341^***^
	(−3.11)	(1.00)	(−3.04)	(−2.73)	(1.00)	(−2.62)
Economic status	3.716^**^	0.400	3.834^**^	1.532	0.400	1.642
	(2.18)	(1.08)	(2.26)	(1.50)	(1.08)	(1.61)
Family history	−0.468	−0.442	−0.599	−0.934	−0.442	−1.055
	(−0.34)	(−1.48)	(−0.44)	(−1.16)	(−1.48)	(−1.31)
_cons	150.7^***^	22.08^***^	157.2^***^	82.83^***^	22.08^***^	88.85^***^
	(57.01)	(38.17)	(39.10)	(55.51)	(38.17)	(33.33)
*N*	918	918	918	918	918	918
*R* ^2^	0.067	0.028	0.071	0.086	0.028	0.095

### 3.4 Urban–rural heterogeneity analysis

In the current social context, the imbalance in urban–rural development is an issue that cannot be overlooked. Urban communities typically have access to more abundant medical and health resources and a higher level of resident health awareness, usually enabling quicker adoption of new technologies and health management knowledge. However, they may also face challenges in health management due to the fast pace of society and high stress levels. In contrast, rural communities often deal with insufficient medical resources and lower health management awareness, where the promotion and use of smart blood pressure monitoring might encounter more obstacles. Nevertheless, the smart blood pressure monitoring intervention project could serve as timely support in these areas, playing a significant role in improving residents’ health management levels. Therefore, the heterogeneity analysis of the smart blood pressure monitoring intervention project between urban and rural settings is crucial. This paper further divides the sample residents into urban and rural community dwellers to analyze the differences in the implementation effects of the smart blood pressure monitoring intervention project.

As indicated in Table 7: rural residents experienced a significant average reduction in systolic blood pressure of 14.85 mm Hg after the intervention compared to the control group, with this result being statistically significant at the 1% level; diastolic blood pressure among rural residents decreased by an average of 6.591 mm Hg after the intervention, also significant at the 1% level; urban residents saw an average reduction in systolic blood pressure of 4.404 mm Hg after the intervention compared to the control group, but this result was not statistically significant (15); however, the average decrease in diastolic blood pressure among urban residents was 6.046 mm Hg, significant at the 1% level.

**Table 7:** Urban–rural heterogeneity analysis.

Variable	(1)	(2)	(3)	(4)
	Rural residents’ systolic blood pressure	Rural residents’ diastolic blood pressure	Urban residents’ systolic blood pressure	Urban residents’ diastolic blood pressure
DID	−14.85^***^	−6.591^***^	−4.404	−6.046^***^
	(−4.01)	(−3.03)	(−1.21)	(−2.72)
Time	1.597	2.694*	−3.844	−1.541
	(0.63)	(1.85)	(−1.58)	(−1.00)
Group	5.664**	2.234	−0.321	0.719
	(2.06)	(1.40)	(−0.12)	(0.41)
Age	−6.464^***^	4.846^***^	−5.910^***^	4.556^***^
	(−2.96)	(3.52)	(−2.72)	(3.19)
Gender	0.497	2.391^**^	2.258	2.369^**^
	(0.26)	(2.02)	(1.11)	(1.97)
Marital status	1.235	0.425	−4.404	2.094
	(0.46)	(0.30)	(−1.58)	(1.41)
Education level	−2.024	1.323	−1.709	−2.392^**^
	(−0.66)	(0.85)	(−0.88)	(−2.06)
Economic status	3.128	−0.718	0.949	1.893
	(1.03)	(−0.41)	(0.46)	(1.49)
Family history	0.557	−0.339	−3.385^*^	−2.397^**^
	(0.30)	(−0.31)	(−1.72)	(−2.00)
_cons	151.4^***^	85.50^***^	153.0^***^	82.57^***^
	(37.45)	(38.92)	(41.87)	(40.74)
*N*	482	482	436	436
*R* ^2^	0.072	0.069	0.061	0.115

Possible reasons include: (1) Resource differences: Due to the relative scarcity of medical resources in rural areas, smart blood pressure monitors, as a convenient health management tool, may better fill the gap in medical services in these areas, especially in providing daily monitoring and health guidance, hence the more pronounced effects in rural areas. (2) Differences in Health Awareness: Urban residents typically possess higher health awareness and self-management skills, possibly engaging in some degree of blood pressure management even before the project’s initiation, thus the additional effects brought by the intervention project may be relatively modest. In contrast, rural residents’ health awareness and self-management capabilities may be lower, making the impact of the intervention project more pronounced. (3) Lifestyle Differences: Urban residents may experience a faster pace of life and greater life stress. Improvements in these lifestyle factors play a crucial role in lowering blood pressure, especially diastolic pressure (16, 17). On the other hand, the lifestyle of rural residents tends to be simpler and more regular, which might be more conducive to the implementation and effectiveness of the intervention project. (4) Acceptance and adherence to intervention: Rural residents may have higher acceptance of new technologies and intervention measures, as this might be one of their primary ways to access health services. In contrast, urban residents may have more options and resources, hence potentially lower adherence to the intervention project. These factors collectively influence the nonsignificant effect of the smart blood pressure monitoring intervention project on reducing systolic blood pressure in urban community residents but significantly lower diastolic pressure in urban residents and more significant reductions in both systolic and diastolic pressures in rural residents (18).

## 4 Discussion

Epidemiological studies have shown a continuous positive correlation between population blood pressure levels and the occurrence of cardiovascular and cerebrovascular death events, with the risk of such mortality events doubling for every 10–20 mm Hg increase in blood pressure (19, 20). Hypertension is closely linked to unhealthy lifestyles, and such “lifestyle diseases” can be effectively managed by establishing healthy living and dietary habits, improving patients’ quality of life, and preventing complications (21, 22). In this study, based on community follow-up management, patients could monitor their blood pressure using smart blood pressure monitors, with monitoring data automatically transmitted in real-time to a cloud management platform and forming blood pressure graphical reports. The platform connects the community doctors managing the patients and the patients themselves. Community doctors can instantly check patients’ past blood pressure data through an app, using it as a basis to provide timely health guidance and consultation related to hypertension. Patients can check their blood pressure trends via a WeChat platform and engage in two-way interactions with their managing doctors.

To better exclude confounding factors and demonstrate the intervention effect, this article uses a Difference-in-Differences (DID) model to analyze the community hypertension management model based on home smart blood pressure monitoring with IoT technology. After adjusting for factors such as age, gender, marital status, education level, economic status, and family history, the implementation of the smart blood pressure monitoring intervention project led to a significant reduction in hypertensive patients’ systolic blood pressure by 9.883 mm Hg and diastolic blood pressure by 6.553 mm Hg compared to changes in the control group (*p* < 0.05), indicating that the community hypertension management model significantly improves the control rate of hypertension among community patients (23). Mediating effect analysis shows that the smart blood pressure monitoring intervention project reduces residents’ blood pressure levels by enhancing the frequency of blood pressure measurements and the attitudes and beliefs toward hypertension treatment (24, 25). The mediating effects of blood pressure measurement frequency, and hypertension treatment attitudes and beliefs account for 5.82 and 8.07% of the total effect, respectively. Urban–rural heterogeneity analysis reveals significant differences under varying resources, health awareness, lifestyles, and acceptance and adherence to intervention: after the intervention, rural residents’ systolic blood pressure significantly decreased by an average of 17.73 mm Hg, and diastolic pressure by 7.002 mm Hg at the 1% statistical level; urban residents’ systolic pressure decreased by an average of 4.775 mm Hg, which was not statistically significant, whereas their diastolic pressure decreased by 5.950 mm Hg, significant at the 1% level.

In summary, the community hypertension management model based on home smart blood pressure monitoring with IoT technology facilitates the timely transmission of blood pressure monitoring data, improves communication between patients and doctors, thereby enhancing the quality of hypertension follow-up management, increasing patients’ awareness of regular blood pressure measurement, strengthening attitudes and beliefs toward hypertension treatment, and consequently improving the control rate of hypertension among community patients. Clinically, this suggests that integrating such technology could lead to substantial improvements in blood pressure control among hypertensive patients, potentially reducing the risk of hypertension-related complications and improving overall patient outcomes. The study particularly highlights the effectiveness of this technology in rural areas, where healthcare resources and patient monitoring may be less frequent and robust compared to urban settings. This could guide policy decisions and healthcare strategies, promoting the wider adoption of IoT technologies in managing chronic diseases like hypertension, and supporting the shift toward more proactive and personalized healthcare solutions.

Based on the findings of this study, the following three recommendations are proposed: First, advance the deep integration and widespread application of smart blood pressure monitoring technology. Medical institutions and government departments should accelerate the technological innovation and popularization of smart blood pressure monitors, especially in rural areas, to optimize the health management system for hypertension patients. By constructing an interconnected smart blood pressure monitoring network that enables real-time data sharing and analysis, more accurate and personalized treatment plans can be provided to patients. This approach aims to enhance the control rate of hypertension and reduce the occurrence of cardiovascular and cerebrovascular complications. Second, cultivate the health management awareness and capabilities of hypertension patients. It is necessary to conduct targeted health education activities at the community level, strengthening the cultivation of treatment attitudes, beliefs, and self-monitoring abilities among hypertension patients. Through multi-channel and varied forms of publicity and education, patients’ awareness of hypertension can be improved. Encouraging active participation in blood pressure management, developing healthy lifestyle habits, and regular blood pressure monitoring can effectively control blood pressure levels and improve quality of life. Third, develop differentiated hypertension management strategies for different regions. Considering the differences in resource access, health awareness, and lifestyles between urban and rural patients, governments and medical institutions should formulate hypertension management plans that are tailored to the local context. In urban areas, focus should be placed on managing psychological stress and lifestyle interventions. In contrast, in rural areas, efforts should be directed toward enhancing the accessibility of medical resources and patients’ health management awareness. This approach aims to achieve precise management and effective intervention for hypertension patients, thereby further improving the overall effectiveness of hypertension management.

## 5 Research limitations

Although this study extensively explored the improvements and mechanisms of community hypertension management models based on home smart blood pressure monitoring with IoT technology, future research in several areas is warranted. First, an evaluation of the economic impact of widespread adoption of this technology could be conducted, assessing cost-effectiveness in terms of healthcare savings, reduced hospital visits, and the economic benefits of enhanced patient quality of life. Such economic analysis would provide compelling reasons for healthcare providers and policymakers to invest in this technology. Additionally, another promising research avenue involves investigating the integration of these IoT systems with other health management tools and platforms to create a more comprehensive approach to chronic disease management. Such studies would offer deeper insights into the scalability and sustainability of smart health interventions across diverse healthcare systems and patient populations. By exploring these research directions, the scientific community can better understand the full potential of IoT technologies in managing hypertension and other chronic diseases, ultimately leading to more effective and proactive healthcare solutions globally.

## Data availability statement

The datasets presented in this study can be found in online repositories. The names of the repository/repositories and accession number(s) can be found in the article/Supplementary material.

## Ethics statement

The studies involving humans were approved by the Medical Ethics Committee of the Centre for Health Management and Policy Research at Shandong University (Approval No. ECSHCMSDU20220101). The studies were conducted in accordance with the local legislation and institutional requirements. The participants provided their written informed consent to participate in this study. Written informed consent was obtained from the individual(s) for the publication of any potentially identifiable images or data included in this article.

## Author contributions

CL: Conceptualization, Data curation, Formal analysis, Funding acquisition, Investigation, Methodology, Project administration, Resources, Software, Supervision, Validation, Visualization, Writing – original draft, Writing – review & editing. SF: Data curation, Investigation, Methodology, Software, Visualization, Writing – original draft. HL: Conceptualization, Data curation, Formal analysis, Funding acquisition, Investigation, Methodology, Project administration, Resources, Software, Supervision, Validation, Visualization, Writing – review & editing.
